# The Cyprus Women’s Health Research (COHERE) initiative: normative data from the SF-36v2 questionnaire for reproductive aged women from the Eastern Mediterranean

**DOI:** 10.1007/s11136-022-03100-7

**Published:** 2022-02-14

**Authors:** B. Swift, H. Naci, B. Taneri, C. M. Becker, K. T. Zondervan, N. Rahmioglu

**Affiliations:** 1 Oxford Endometriosis CaRe Centre, Nuffield Department of Women’s and Reproductive Health, University of Oxford, Oxford, OX3 9DU UK; 2 Wellcome Centre for Human Genetics, University of Oxford, Oxford, OX3 7BN UK; 3 Department of Health Policy, London School of Economics and Political Science, London, WC2A 2AE UK; 4 Department of Biological Sciences, Faculty of Arts and Sciences, Eastern Mediterranean University, Famagusta, Northern Cyprus; 5 Department of Genetics and Cell Biology, Faculty of Health, Medicine & Life Sciences, Institute for Public Health Genomics, Maastricht University, Maastricht, The Netherlands

**Keywords:** SF-36, Health-related quality of life, Northern Cyprus, Population norms, Women’s health, Patient-reported outcome (PRO)

## Abstract

**Purpose:**

Describe the health-related quality of life for a representative cohort of women aged 18–55 in Northern Cyprus.

**Methods:**

We utilised the SF-36-Health-Survey-version-2 (SF-36v2) questionnaire as part of the COHERE Initiative study to calculate the eight physical and mental subscale scores, as well as the two overall summary measures for physical and mental health, where we present results using Cyprus-specific scoring as well as scores based on the test developers’ algorithms. We examined associations between sociodemographic characteristics for both scores.

**Results:**

A total of 7089 women fully completed the SF-36v2 questionnaire (mean age = 36.9), which was reliable and valid in this population. We observed better physical health in ages 18–25 compared to 46–55 (53.32 vs. 46.72 (*p* < 0.001)) and better mental health in women aged 46–55 compared to 18–25 (52.07 vs. 47.95 (*p* < 0.001)). Women in employment had better physical and mental health compared to those who were unemployed (physical: 50.25 vs 49.95, *p* < 0.001 and mental: 50.25 vs 49.24, *p* = 0.083) and scores increased as educational attainment increased (physical: 47.55 for primary to 51.58 for postgraduate, mental: 48.88 to 50.59, *p* < 0.001). Turkish Cypriot women had higher scores than Turkish women (physical: 50.42 vs 49.30, mental: 50.43 vs 49.10, *p* < 0.001).

**Conclusion:**

These are the first population normative values published from a large representative sample of women between 18 and 55 years from the Eastern Mediterranean region. We found better physical health in younger women and better mental health in older women. Turkish Cypriot women and non-migrant women had better mental health, and HRQOL was highest in those in paid employment and those with a higher educational achievement.

**Supplementary Information:**

The online version contains supplementary material available at 10.1007/s11136-022-03100-7.

## Introduction

Health-related quality of life (HRQOL) is important when exploring the general wellbeing of the population, as well to evaluate specific health states. The Short Form-36 Health Survey (SF-36) is a self-reported multi-dimensional measure widely used across countries, with its use ranging from monitoring the burden of disease, to examining the cost-effectiveness of a treatment [1]. In order to aid interpretation of the data, the developers recommend that a normative-based scoring method using US weights is used to provide a standard with which scores from other populations can be compared. However, there is much discussion surrounding whether it is appropriate to use weights that may not be culturally specific, especially when there are differences in health states.

Cyprus is the third largest Mediterranean island with around 300,000 Turkish Cypriot and 700,000 Greek Cypriot residents. There is a lack of population-level health data from Northern Cyprus due to unresolved political circumstances [2, 3] and as a result, this part of the island is absent from any published health statistics from the region. To date, there are no published studies that have provided population normative values in Cyprus for the SF-36, and it is inappropriate to use published values from other countries given the differences in healthcare. A cross-sectional study in Limassol, Republic of Cyprus examined HRQOL in the region [4], but it did not provide normative values for the island and instead used normative values derived from the U.S.A. Another cross-sectional study in Izmir, Turkey, used the SF-36 to provide population norms for the urban population [5]. However, the study took part just after a major economic crisis which is likely to have had an impact on the mental health component of the quality of life scores.

This paper seeks to address the lack of normative data applicable to Northern Cyprus and also serves to add further reference values for the Eastern Mediterranean region. We use data from the Cyprus Women’s Health Research (COHERE) Initiative, to provide population norms for the eight SF-36-Health-Survey version 2 (SF-36v2) health domains as well as the two higher-order summary scores; Physical Component Summary (PCS) and Mental Component Summary (MCS). We examine the reliability and the validity of this dataset and its ability to provide this information, as well as testing the construct validity by investigating statistical relationships between each domain and a range of demographic variables that are known to be related to health outcomes including age, ethnicity, migration status, educational attainment, region of residence, civil status and employment status.

## Methods

### Study sample: the COHERE Initiative

Normative values for the SF-36v2 and the physical and mental health scoring coefficients were estimated using data collected as part of the COHERE Initiative. The COHERE Initiative is a population based cross-sectional study that has recruited 7646 consenting women between the ages of 18–55 in Northern Cyprus. The aim of COHERE is to determine the relative burden of women’s health conditions and related co-morbidities in women living in Northern Cyprus and as such, establish a women’s health cohort for future follow-up. In short, each participant completed the baseline questionnaire—an expanded version of the Endometriosis-Phenome-and-Biobanking-Harmonization-Project (EPHect) questionnaire [6] which included the SF-36v2 generic health measurement. Data were collected through a combination of household (16%, (*n* = 1208)) and workplace (84% (6438)) face-to-face visits (93% (7128)) as well as through online (7% (518)) recruitment methods [7], between January 2018 and February 2020. Women aged between 18 and 55 at recruitment, who were either citizens of Northern Cyprus or had been residing there for the past 5 years and were able to give informed consent were eligible to participate in the study. Women were recruited into the study from the 6 main districts in Northern Cyprus (Nicosia, Kyrenia, Famagusta, Morphou, Trikomo and Lefke) with recruitment targets being set using geographic population densities.

We compared the age, educational attainment, civil status, employment status, and city of residence structure of our sample with the projected 2019 population figures for Northern Cyprus obtained from the Northern Cyprus Statistics Institution (available on request from: http://www.stat.gov.ct.tr/). Our sample was broadly representative of these projected values with the main differences being seen in age and education. We calculated weights for age and education and present all SF-36v2 scores obtained from the sample both unweighted and weighted.

### Ethics

The study was approved by the Oxford Tropical Research Ethics Committee (OxTREC) of the University of Oxford (OxTREC reference: 37–17). The study also received local ethics approval from the Eastern Mediterranean University Ethics Committee (ETK00-2017-0240).

### SF-36v2 health domain subscales

The SF-36 has been validated previously in the Turkish language [8, 9]. Within the SF-36, there are 36 items that measure 8 domains as follows: limitations in physical activity due to health problems (physical functioning, 10 items), limitations in social activities due to physical or emotional problems (social functioning, 2 items), limitations in usual role activities due to physical health problems (role physical, 4 items), limitations in usual role activities due to emotional problems (role emotional, 3 items), wellbeing and psychological distress (mental health, 5 items), energy and fatigue (vitality, 4 items), bodily pain (bodily pain, 2 items) and perceptions of general health (general health, 5 items). Within the 36 items, there is a singular additional question asking about changes in health over the past year. Using the methods set out by Ware et al*.* [10, 11]*,* the items within each of the above dimensions were coded, summed, and transformed (calculated by subtracting the lowest possible raw score from the actual raw score, dividing by the possible raw score range, and multiplying this by 10. This gave a scale from 0 (worst possible health state as measured by the questionnaire) to 100 (best possible health state).

We also calculated the 8 health domains using norm-based scoring using a T-score transformation where the mean was set to 50 and the standard deviation to 10 in the current sample.

### SF-36v2 component summary scores

Following the methods set out in the SF-36v2 manual, the data were factor analysed to produce scoring coefficients for PCS and MCS [11]. In short, we used principal components analysis to produce factor loadings, applied an orthogonal varimax rotation to rotate these loadings and then obtained scores. Calculating PCS involved multiplying each SF-36 scale z-score (calculated by subtracting the mean of SF-36 scale and dividing the difference by the corresponding scale standard deviation) by its respective factor score coefficient and in the case of the MCS, this involved multiplying each SF-36 scale z-score by its respective factor score coefficient. Finally, a T-score transformation was used to standardise the scores whereby the mean was set to 50 and the standard deviation to 10. PCS and MCS are two clusters that are produced from each of the eight scales and allow for further interpretation of the results.

Although no previous studies have investigated the SF-36v2 measures in Northern Cyprus, a cross-cultural adaptation of the survey was shown to be successful in Turkey and demonstrated an acceptable level of reliability and validity in its use in Turkish speaking individuals [5, 8]. To investigate whether the SF-36v2 questionnaire was valid in Northern Cyprus, reliability and validity was assessed. We also calculated summary measures using US-specific health domain subscale scores from 1998 and US-specific factor score coefficients from 1990 [12].

### Reliability

Cronbach’s alpha was used to examine internal consistency reliability; a value of > 0.7 was considered satisfactory. To assess whether the summary measures, PCS and MCS were reliable, the reliability of each of the eight subscales, the covariances among them and the factor score coefficients were calculated.

### Validity

Principal components analysis, item-subscale correlations (item-rest correlations for the subscales and their respective items) and inter-scale correlations (Spearman correlations) were used to assess construct validity. A value of > 0.4 for item-rest correlations was considered satisfactory. If the correlation between an item and the sum of the other items its respective subscale (item-rest correlation) was shown to be significantly higher than its correlation with other subscales (item-subscale correlations) then the item’s inclusion in its subscale was supported. If the correlation between two subscales was less than their reliability coefficients (Cronbach’s alpha), then it can be said that there is evidence of reliable variance measured by the respective subscales.

### Sociodemographic characteristics

We used self-reported data from the COHERE questionnaire in order to obtain potential covariates. Age was calculated by subtracting birthyear from survey completion date and categorised into the following four groups: 18–25, 26–35, 36–45 and 46–55. Ethnicity was split into 3 groups: Turkish Cypriot, Turkish, Mixed/Other (women reporting to have 2 ethnicities and women reporting to be a single ethnicity other than Turkish Cypriot or Turkish). Migration status (born in Northern Cyprus or parents born in Northern Cyprus, not born in Northern Cyprus and therefore recent migrant to the island). Highest educational achievement (primary or middle school, high school or post-secondary, undergraduate and postgraduate), employment status (employed or unemployed), civil status (single, married, divorced/widowed), city of residence (Nicosia, Famagusta, Trikomo, Lefke, Morphou, Kyrenia) were also assessed using the questionnaire data.

### Missing data

As we used a large sample size (*n* = 7646) and the intention of this primary analysis was to obtain the factor loadings to construct the summary scores, data substitution algorithms were not used. In addition to this, there has been speculation that substitution of mean values may have either a conservative or attenuated bias [13]. Given these are the first normative scores to be calculated for women in Northern Cyprus between the ages of 18–55, it is also important to report ‘actual’ scores, so that normative scores here can be used for future analyses and are not differentially attenuated according to the amount of missing data that has been recorded [14]. This approach is consistent with previously published studies [15, 16].

### Statistical analyses

Descriptive statistics for each of the eight subscales for the included sample and different subsamples according to age, ethnicity, migration status, educational achievement, civil status, city of residence and employment status were calculated. Differences in means of the eight health subscales for each of the subscales were tested using linear regression. Regressions were computed both crudely and after adjustment for age, as age is usually correlated with the covariates examined here. Regressions for ethnicity and migration status were additionally adjusted for educational achievement and employment classification, as these two demographics are often correlated with non-native people. Statistical analyses were carried out using Stata SE version 16.0.810 (StataCorp LP, College Station, Texas, USA) and R Studio.

## Results

### Sample population and data completion

Our sample population consists of 7646 women with a mean age of 36.9 years. Women in our cohort were more likely to be Turkish Cypriot (73.8%), native to the island (74.6%) have a university degree (52.7%), be married (66.5%), be in paid employment (81.2%) and reside in the capital city, Nicosia (43.2%) (Table 1). Percentages for whom scale scores could not be calculated due to incomplete items for a particular scale was low and ranged from 0.5 to 2.9% (data not shown).

**Table 1:** Comparison between various demographics of participants within the Cyprus Women's Health Research (COHERE) Initiative and the census for Northern Cyprus

	Characteristics	*N* (%)	
		COHERE	Census
Age at study (years)	18–25	1108 (14.5)	22,355 (22.7)
	26–35	2383 (31.2)	31,228 (31.6)
	36–45	2447 (32.0)	25,318 (25.7)
	46–55	1708 (22.3)	18,784 (20.0)
Education	Primary or middle school	845 (11.8)	28,426 (33.4)
	High school or post-secondary	2557 (35.6)	31,748 (37.3)
	Undergraduate	2583 (35.9)	19,713 (23.2)
	Postgraduate	1204 (16.8)	5206 (6.1)
Civil status	Single	1753 (24.1)	24,015 (23.8)
	Married	4839 (66.5)	68,257 (67.5)
	Divorced/separated or widowed	684 (9.4)	8809 (8.7)
Employment^a^	In paid employment	5929 (81.2)	46,089 (91.1)
	Not in paid work	1374 (18.8)	871 (1.9)
City of residence	Famagusta	1611 (21.1)	25,300 (25.6)
	Kyrenia	1263 (16.5)	23,848 (24.2)
	Lefke	2691 (3.7)	261 (3.4)
	Morphou	585 (7.7)	6095 (6.2)
	Nicosia	3301 (43.2)	32,503 (32.9)
	Trikomo	625 (8.2)	7249 (7.3)

Only includes women between the ages 18–55. Ethnicity and migration status were not available

^a^In paid employment includes both employees and self-employed. Not in paid work includes students, homemakers, retirees, and those without employment

### Validation of the SF-36v2 questionnaire for women in Northern Cyprus

Regarding reliability, Cronbach’s alpha coefficients were found to be satisfactory (> 0.70) for all health domains (Table 2), apart from general health (0.69). Reliability of the summary measures was 0.89 for both PCS and MCS.

**Table 2:** SF-36v2 health domain subscales: mean 0–100 scores with 95% confidence intervals, standard deviation, percentage floor, percentage ceiling, factor score coefficients, and Cronbach's alpha

Scale	*N*	Mean 0–100 score	95% CI	SD	Percentage floor (%)	Percentage ceiling (%)	Factor score coefficients		Cronbach's alpha
							PCS	MCS	
Physical functioning (PF)	7607	88.28	(87.92, 88.63)	15.84	0.17	39.49	0.49	− 0.21	0.87
Role Physical (RP)	7558	80.67	(80.15, 81.18)	22.81	0.48	41.81	0.41	− 0.11	0.92
Bodily pain (BP)	7596	68.22	(67.67, 68.77)	24.38	1.20	22.63	0.35	− 0.08	0.87
General health (GH)	7548	63.79	(63.35, 64.24)	19.82	0.21	1.50	0.17	0.08	0.69
Vitality (VT)	7426	57.50	(57.03, 57.97)	20.71	0.83	1.25	− 0.15	0.37	0.74
Social functioning (SF)	7577	77.40	(76.87, 77.92)	23.27	0.73	35.87	0.03	0.22	0.74
Mental health (MH)	7455	64.05	(63.60, 64.50)	19.81	0.31	1.99	− 0.20	0.41	0.83

*CI* confidence interval, *SD* standard deviation, *PCS* physical component summary, *MCS* mental component summary

When looking at validity, item-rest correlations were all satisfactory apart from PF10 of the physical functioning perception subscale (0.38). The principal components analysis produced two factors with eigenvalue > 1 which indicates a two-factor structure. All items were satisfactory when looking at differences between item-rest correlations and inter-scale correlations i.e. correlations were higher between individual items and their respective subscales, than between individual items and the other 7 subscales. It was also found that the correlations between subscales were lower than their respective Cronbach’s alpha values which suggest that there is unique reliable variance (Table 2). Ceiling effects were highest in physical functioning and role physical subscales (39–42%) with the bodily pain subscale having the highest floor effect (1.20%).

### Health domain subscales

Women who were younger had better physical health (PF, RP, BP) compared to older women but as age increased, mean scores for the mental health subscales also increased (VT, RE, MH) (Table 3). Though GH increased with age, this was not statistically significant. When considering ethnicity, women who self-reported to be Turkish had the lowest scores across all domains except MH, RE, SF, and all but PF remained statistically significant after adjustment for age (Table 4). Women who were not migrants had higher subscale scores for all the domains compared to women with a migration background (PF, BP, GH, VT, SF, RE, MH (Supplementary Table 2). After adjustment for age, married women generally had the best mental health (MH, RE, SF) but the worst physical health (PF, RP, BP) (Supplementary Table 3). Residency of the women in COHERE did not appear to significantly affect physical or mental health domains (Supplementary Table 4). As educational attainment increased, mean physical health also increased after adjustment for age, (PF, RP, BP, GH) as did mental health domains (VT, RE and MH) (Table 5). Generally, women who were employed had significantly better physical subscale scores (PF, RP, BP, GH) with only the VT mental health score remaining significant after adjustment for age (Table 6).

**Table 3:** SF-36v2 health domain subscales: mean 0–100 scores with 95% confidence intervals, standard deviation, *p* values from linear regression (global test) according to age (18–25, 26–35, 36–45, 46–55)

Scale	18–25 years				26–35 years				36–45 years			
	*N*	Mean 0–100 score	95% CI	SD	*N*	Mean 0–100 score	95% CI	SD	*N*	Mean 0–100 score	95% CI	SD
Physical functioning (PF)	1107	93.23	(92.50, 93.97)	12.46	2374	90.97	(90.42, 91.53)	13.77	2431	87.90	(87.28, 88.52)	15.61
Role Physical (RP)	1103	85.17	(83.94, 86.41)	20.93	2361	82.09	(81.19, 82.99)	22.30	2407	79.50	(78.58, 80.41)	22.84
Bodily pain (BP)	1100	72.07	(70.64, 73.49)	24.08	2370	69.93	(68.98, 70.89)	23.71	2426	67.28	(66.31, 68.25)	24.39
General health (GH)	1098	63.41	(62.22, 64.60)	20.07	2361	64.35	(63.57, 65.13)	19.32	2413	62.87	(62.07, 63.68)	20.18
Vitality (VT)	1086	56.19	(54.95, 57.43)	20.84	2315	57.35	(56.54, 58.17)	20.08	2372	57.18	(56.34, 58.02)	20.86
Social functioning (SF)	1100	77.32	(75.95, 78.68)	23.07	2363	76.78	(75.82, 77.73)	23.74	2420	77.71	(76.80, 78.61)	22.75
Role emotional (RE)	1093	73.73	(72.22, 75.23)	25.33	2333	76.79	(75.82, 77.75)	23.85	2379	77.78	(76.83, 78.72)	23.50
Mental health (MH)	1085	62.09	(60.87, 63.32)	20.57	2339	63.91	(63.13, 64.69)	19.30	2370	63.73	(62.94, 64.53)	19.71

Scale	46–55 years				*p* value
	*N*	Mean 0–100 score	95% CI	SD	
Physical functioning (PF)	1695	81.81	(80.92, 82.69)	18.50	** < 0.001**
Role physical (RP)	1687	77.39	(76.25, 78.54)	23.98	** < 0.001**
Bodily pain (BP)	1700	64.68	(63.50, 65.87)	24.92	** < 0.001**
General health (GH)	1676	64.58	(63.63, 65.53)	19.76	0.665
Vitality (VT)	1653	59.03	(58.01, 60.05)	21.21	**0.001**
Social functioning (SF)	1694	77.88	(76.76, 79.00)	23.47	0.146
Role emotional (RE)	1652	78.80	(77.68, 79.93)	23.25	** < 0.001**
Mental health (MH)	1661	65.99	(65.03, 66.95)	19.98	** < 0.001**

*N* total number *CI* confidence interval, *SD* standard deviation

*p* values from linear regression (crude)

*p* values < 0.05 are bold

**Table 4:** SF-36v2 health domain subscales: mean 0–100 scores with 95% confidence intervals, standard deviation, *p* values from linear regression (global test) without and with adjustment for age according to ethnicity (Turkish Cypriot, Turkish, Other/Mixed)

Scale	Turkish Cypriot				Turkish			
	*N*	Mean 0–100 score	95% CI	SD	*N*	Mean 0–100 score	95% CI	SD
Physical functioning (PF)	5359	88.71	(88.30, 89.13)	15.52	1516	87.30	(86.45, 88.15)	16.91
Role physical (RP)	5323	81.26	(80.67, 81.86)	22.23	1507	79.69	(78.46, 80.93)	24.40
Bodily pain (BP)	5346	69.40	(68.76, 70.04)	23.79	1516	64.72	(63.40, 66.03)	26.09
General health (GH)	5317	64.61	(64.08, 65.13)	19.60	1501	61.54	(60.49, 62.59)	20.75
Vitality (VT)	5238	58.53	(57.99, 59.08)	20.21	1472	54.38	(53.26, 55.51)	22.04
Social functioning (SF)	5335	78.13	(77.53, 78.74)	22.58	1512	76.58	(75.33, 77.83)	24.77
Role emotional (RE)	5260	77.94	(77.32, 78.57)	23.22	1480	75.72	(74.42, 77.02)	25.45
Mental health (MH)	5261	64.86	(64.34, 65.38)	19.22	1481	62.29	(61.22, 63.37)	21.14

Scale	Other/mixed				*p* value	
	*N*	Mean 0–100 score	95% CI	SD	Crude	Adjusted for age
Physical functioning (PF)	383	89.92	(88.66, 91.19)	12.63	0.362	0.116
Role physical (RP)	384	80.32	(78.03, 82.62)	22.96	**0.042**	**0.020**
Bodily pain (BP)	384	67.21	(64.72, 69.69)	24.85	** < 0.001**	** < 0.001**
General health (GH)	386	63.85	(61.95, 65.75)	19.04	** < 0.001**	** < 0.001**
Vitality (VT)	381	55.61	(53.40, 57.82)	22.01	** < 0.001**	** < 0.001**
Social functioning (SF)	386	73.45	(70.87, 76.02)	25.83	** < 0.001**	** < 0.001**
Role emotional (RE)	379	74.85	(72.38,77.31)	24.51	** < 0.001**	** < 0.001**
Mental health (MH)	379	61.46	(59.24, 63.69)	22.13	** < 0.001**	** < 0.001**

*N* total number *CI* confidence interval, *SD* standard deviation

*p* values from linear regression (crude) and with adjustment for age (adjusted for age)

*p* values < 0.05 are bold

**Table 5:** SF-36v2 health domain subscales: mean 0–100 scores with 95% confidence intervals, standard deviation, *p* values from linear regression (global test) without and with adjustment for age according to educational attainment (Primary or middle school, high school or post-secondary, undergraduate degree, postgraduate degree)

Scale	Primary or middle school				High school or post-secondary			
	*N*	Mean 0–100 score	95% CI	SD	*N*	Mean 0–100 score	95% CI	SD
Physical functioning (PF)	838	84.18	(82.87, 85.48)	19.29	2538	87.23	(86.59, 87.87)	16.53
Role physical (RP)	834	77.63	(75.80, 79.46)	26.97	2516	80.00	(79.09, 80.91)	23.34
Bodily pain (BP)	843	61.37	(59.47, 63.28)	28.24	2537	67.43	(66.46, 68.40)	24.87
General health (GH)	831	58.42	(56.96, 59.88)	21.44	2516	63.45	(62.65, 64.26)	20.67
Vitality (VT)	820	51.27	(49.69, 52.84)	23.01	2467	57.14	(56.27, 58.01)	22.03
Social functioning (SF)	842	76.68	(74.92, 78.44)	26.03	2526	77.39	(76.47, 78.31)	23.55
Role emotional (RE)	816	77.15	(75.37, 78.94)	26.00	2478	76.70	(75.75, 77.65)	24.14
Mental health (MH)	821	61.19	(59.64, 62.73)	22.58	2486	63.44	(62.62, 64.25)	20.70

Scale	Undergraduate degree				Postgraduate degree				*p* value	
	*N*	Mean 0–100 score	95% CI	SD	*N*	Mean 0–100 score	95% CI	SD	Crude	Adjusted for age
Physical functioning (PF)	2579	90.27	(89.75, 90.79)	13.56	1203	90.54	(89.74, 91.34)	14.12	** < 0.001**	** < 0.001**
Role physical (RP)	2570	81.96	(81.15, 82.77)	20.99	1197	82.84	(81.63, 84.05)	21.36	** < 0.001**	** < 0.001**
Bodily pain (BP)	2570	69.94	(69.06, 70.83)	22.95	1196	71.55	(70.27, 72.82)	22.44	** < 0.001**	** < 0.001**
General health (GH)	2566	64.86	(64.14, 65.58)	18.63	1193	66.79	(65.72, 67.85)	18.76	** < 0.001**	** < 0.001**
Vitality (VT)	2525	58.85	(58.09, 59.60)	19.32	1183	59.95	(58.89, 61.00)	18.49	** < 0.001**	** < 0.001**
Social functioning (SF)	2566	77.84	(76.97, 78.71)	22.38	1198	78.01	(76.74, 79.27)	22.37	0.162	0.094
Role emotional (RE)	2540	77.54	(76.65, 78.44)	23.07	1189	78.30	(77.02, 79.58)	22.58	0.095	**0.012**
Mental health (MH)	2535	65.02	(64.30, 65.74)	18.46	1182	65.79	(64.75, 66.83)	18.22	** < 0.001**	** < 0.001**

*N* total number *CI* confidence interval, *SD* standard deviation

*p* values from linear regression (crude) and with adjustment for age (adjusted for age)

*p* values < 0.05 are bold

**Table 6:** SF-36v2 health domain subscales: mean 0–100 scores with 95% confidence intervals, standard deviation, *p* values from linear regression (global test) without and with adjustment for age according to employment status (In paid employment, not in paid employment)

Scale	In paid employment				Not in paid employment				*p* value	
	*N*	Mean 0–100 score	95% CI	SD	*N*	Mean 0–100 score	95% CI	SD	Crude	Adjusted for age
Physical functioning (PF)	5901	88.49	(88.10, 88.88)	15.26	1370	88.28	(87.36, 89.21)	17.50	0.659	** < 0.001**
Role physical (RP)	5865	80.97	(80.40, 81.53)	21.98	1362	80.51	(79.15, 81.87)	25.67	0.506	**0.029**
Bodily pain (BP)	5890	68.56	(67.96, 69.17)	23.62	1368	67.33	(65.87, 68.80)	27.58	0.093	**0.003**
General health (GH)	5860	64.58	(64.09, 65.08)	19.34	1357	61.12	(59.97, 62.28)	21.63	** < 0.001**	** < 0.001**
Vitality (VT)	5762	58.19	(57.67, 58.72)	20.21	1343	54.79	(53.57, 56.01)	22.81	** < 0.001**	** < 0.001**
Social functioning (SF)	5878	77.33	(76.74, 77.92)	23.05	1367	78.45	(77.17, 79.72)	24.09	0.110	0.066
Role emotional (RE)	5780	77.64	(77.04, 78.24)	23.25	1351	75.98	(74.60, 77.36)	25.84	0.021	0.127
Mental health (MH)	5781	64.32	(63.82, 64.82)	19.40	1352	63.44	(62.29, 64.59)	21.54	0.143	0.430

In paid employment includes both employees and self-employed. Not in paid work includes students, homemakers, retirees, and those without employment

*N* total number *CI* confidence interval, *SD* standard deviation

*p* values from linear regression (crude) and with adjustment for age (adjusted for age)

*p* values < 0.05 are bold

The 8 domains presented as norm-based scores can be seen in the Appendix (Supplementary Tables S4-S10).

### SF-36v2 summary measures PCS and MCS

Principal components analysis and orthogonal rotation was used to factor analyse the data and led to a two-factor solution; factor labelled PCS gained an eigenvalue of 4.00, with MCS having an eigenvalue of 1.14. We found better physical health (PCS) in younger women and generally better mental health in older women (Table 7). Higher scores were seen in women with a higher educational achievement and those in paid employment. Single women appeared to have the best physical health and the worst mental health (mean age in single women = 27.03 (SD 7.20) vs married women = 39.74 (SD 8.16)). Women residing in Morphou had the lowest PCS scores and those in Famagusta the worst MCS scores, with women in Kyrenia having the highest PCS scores and those in Lefke the highest MCS scores. These associations were not significant once adjusting for age (mean age and SD in each district as follows: Famagusta (mean = 35.5 (SD 9.8)), Kyrenia (mean = 37.2 (SD 9.4)), Lefke (mean = 38.1 (SD 9.5)), Morphou (mean = 37.6 (SD 10.2)), Nicosia (mean = 37.4 (SD 9.3)) and Trikomo (mean = 36.1 (SD 10.3)) (Table 7). Turkish Cypriot women had both the best physical (with other/mixed ethnicities) and mental health scores after adjustment for age as did those women with a non-migration background. After further adjustment for education and occupation classification, associations were especially attenuated for migration status and health scores and although the effect was also lessened between ethnicity and the two health scores, associations remained significant for  MCS (not shown).

**Table 7:** PCS, MCS: mean (T-scores) with 95% CI, SD and *p* values from linear regression (global test) without and with adjustment for age for the overall sample (*n* = 7089) and subsamples according to age, ethnicity, educational achievement, civil status, city of residence, employment status^*^

Characteristics	PCS			*p* value		MCS			*p* value	
	Mean T-score	95% CI	SD	Crude	Adjusted for age	Mean T score	95% CI	SD	Crude	Adjusted for age
Overall sample	50.10	(49.87, 50.34)	9.97	–	–	50.01	(49.78, 50.25)	10.01	–	–
Age				<0.001					<0.001	
18–25	53.32	(52.82, 53.83)	8.34		–	47.95	(47.31, 48.59)	10.50		–
26–35	51.50	(51.12, 51.87)	9.10			49.43	(49.02, 49.84)	9.97		
36–45	49.58	(48.17, 50.00)	10.01			50.13	(49.72, 50.53)	9.86		
46–55	46.72	(46.18, 47.26)	10.96			52.07	(51.59, 52.54)	9.56		
Ethnicity				0.072	0.014				<0.001	<0.001
Turkish Cypriot	50.42	(50.15, 50.69)	9.76			50.43	(50.16, 50.70)	9.76		
Turkish	49.30	(48.74, 49.86)	10.71			49.10	(48.55, 49.66)	10.59		
Other/mixed	50.82	(49.88, 51.76)	9.05			48.40	(47.26, 49.55)	11.06		
Educational achievement				** < 0.001**	** < 0.001**				** < 0.001**	** < 0.001**
Primary or middle school	47.55	(46.70, 48.40)	12.17			48.88	(48.14, 49.63)	10.64		
High school or post-secondary	49.60	(49.18, 50.01)	10.26			49.95	(49.53, 50.37)	10.42		
University	51.04	(50.69, 51.39)	8.84			50.24	(49.86, 50.63)	9.57		
Postgraduate	51.58	(51.05, 52.11)	9.13			50.59	(50.03, 51.14)	9.59		
Civil status				** < 0.001**	** < 0.001**				** < 0.001**	**0.047**
Single	52.70	(52.28, 53.12)	8.77			48.61	(48.10, 49.11)	10.53		
Married	49.21	(48.91, 49.51)	10.10			50.64	(50.35, 50.92)	9.69		
Divorced/separated or widowed	50.68	(49.87, 51.48)	10.41			49.80	(48.97, 50.62)	10.65		
Migration status				** < 0.001**	**0.002**				** < 0.001**	** < 0.001**
Non migration status	50.48	(50.22, 50.75)	9.70			50.33	(50.06, 50.61)	9.87		
Migration status	49.39	(48.89, 49.90)	10.56			49.18	(48.68, 49.68)	10.47		
City of residence				**0.019**	0.182				0.243	0.578
Famagusta	50.43	(49.93, 50.94)	9.90			49.60	(49.08, 50.11)	10.11		
Kyrenia	50.62	(50.07, 51.16)	9.59			50.04	(49.46, 50.62)	10.12		
Lefke	49.86	(48.63, 51.08)	9.68			51.37	(50.20, 52.53)	9.19		
Morphou	49.14	(48.24, 50.04)	10.83			50.20	(49.33, 51.07)	10.48		
Nicosia	50.08	(49.74, 50.42)	9.68			50.03	(49.68, 50.38)	9.87		
Trikomo	49.38	(48.45, 50.31)	11.45			50.20	(49.38, 51.02)	10.05		
Employment status^a^				0.317	** < 0.001**				**0.001**	0.083
In paid employment	50.25	(50.00, 50.50)	9.50			50.25	(49.99, 50.51)	9.87		
Not in paid work	49.95	(49.32, 50.58)	11.59			49.24	(48.66, 49.82)	10.65		

*PCS* Physical Component Summary *MCS* Mental Component Summary, *CI* confidence interval *SD* standard deviation

P values from linear regression (crude) and with adjustment for age (adjusted for age)

P values < 0.05 are bold

^a^In paid employment includes both employees and self-employed. Not in paid work includes students, homemakers, retirees, and those without employment

After weighting our data by age and education we did not see great differences in the results (Supplementary Tables S11-S19).

PCS estimates calculated using US coefficients were broadly similar to those generated using our sample; however, we found that the MCS estimates were substantially lower (Supplementary Tables S20-S21*).*

## Discussion

We have shown that the SF-36v2 questionnaire is both reliable and valid in women between the ages of 18–55 residing in Northern Cyprus in evaluating HRQOL. We found better physical health in younger women and better mental health in older women. Turkish Cypriot women and non-migrant women had both better mental and physical health, and HRQOL was highest in those in paid employment as well as in those with a higher educational achievement. Here we have provided the first normative values for women in Northern Cyprus aged between 18 and 55 and our analysis suggests that choosing culturally specific weighting coefficients is important when investigating HRQOL.

### Reliability and validity

Internal consistency reliability of the scales was high (above 0.8) for 5 of the eight scales with the general health scale being the only one to fall somewhat short of the accepted Cronbach’s alpha level of 0.7 (0.69). However, we believe this to be sufficiently close to argue that this scale too had adequate internal consistency reliability. The highest ceiling value in our sample was 41.8%, for the role physical scale, suggesting that the SF-36v2 was well interpreted and suited to our population of Northern Cyprus.

### Physical and mental health domains

This study has shown that the highest score of the eight health domains for participants in COHERE was physical functioning and the lowest was vitality. This pattern is consistent with various other studies conducted in a number of high-income countries such as the United Kingdom [16], Switzerland [17] and the United States [11], as well as countries within the Mediterranean region such as Turkey [5] and Greece [18]. Although the scores we present here vary from those presented by other countries, this does not necessarily mean that there are international health differences; there are a number of reasons normative scores may differ between countries, such as differences in culture, expectation of health and mode of administration of the questionnaire.

We observed that as age increased, mean physical health decreased and mean mental health increased, as seen in various other studies [11, 16, 17]. Education and employment are two demographics that can be used as proxies for the sociodemographic position of people within society. As our results showed higher mean scores in those who had obtained higher educational qualifications as well as in those who were in paid employment, we can be confident that the SF-36v2 was capable of detecting these known differences amongst different socioeconomic positions.

Compared to migrant women, non-migrants had the highest mean scores after adjustment for age only, consistent with previously published studies [17, 19]. Once education and occupation type had been adjusted for, the associations were no longer statistically significant, suggesting the observed associations may be partly explained by social disadvantages that arose from lower socioeconomic status immigrants in this sample. When examining ethnicity, after adjustment for age, education, and occupation class, the statistically significant association between ethnicity and mental health remained, with Turkish Cypriot women having the highest mean scores. Most studies examining HRQOL in non-natives show that they suffer from higher stressors when compared to their native counterparts, not only due to differences in socioeconomics, but due to other migration-specific difficulties [20, 21].

The area most similar to Northern Cyprus both geographically and culturally to have produced normative values for the SF-36v2 is Turkey [5]. Mean health domain scores in COHERE were lower for all scales, apart from physical functioning (88.3 vs 80.6). The study in Turkey was focussed on an urban region with a small sample size (670 women) which is not representative of the population of Turkey. In addition to this, five of the eight scales had a median ceiling score of 100 (perfect) which is unusually high. We believe this is why normative HRQOL scores should be both country and culturally specific.

## Strengths and limitations

The cross-sectional design of our study means that we cannot infer causality of measured variables. Gandek and Ware [22] recommend that the minimum sample size used to produce country specific normative values for HRQOL using the SF-36v2 should be between 2500 and 3000 respondents. Here our large sample size (*n* = 7646) means we can be confident in the accuracy of our values and in addition to this, the demographic and social background characteristics of participants in our sample are broadly representative of women between the ages of 18–55 in Northern Cyprus. However, there is some over-representation of women with university degrees and in employment compared to the general population and this should be considered when carrying out any further analyses.

Although the SF-36v2 is a self-administered questionnaire and therefore may suffer from reporting bias, it is reliable and valid as well as being a widely used tool to assess HRQOL. Our study is unique in that it provides normative values for a female population. It is well established that HRQOL is significantly lower in women compared to men and therefore using female-specific normative values from a population of premenopausal women is especially advisable when investigating the impact of women’s reproductive diseases, such as endometriosis, on HRQoL. The main aim of the COHERE Initiative is to investigate reproductive health conditions and so our study is restricted to women between the ages of 18–55. Therefore, it is not generalisable to women above and below these ages or to men and so further research in these groups would be needed to discover if similar patterns exist and to provide normative values for all age groups.

## Conclusion

Here we present the normative values for women aged 18–55 in Northern Cyprus using the SF-36v2 questionnaire. In accordance with the literature, we saw higher mean physical health scores in younger women and higher mean mental health scores in older women. Non-Turkish Cypriot women and migrants had lower mean scores for both domains, as did those who had lower educational attainments and those who were not in paid employment. This research will allow future studies to measure HRQOL as assessed by the SF-36v2 questionnaire using female-specific data in Northern Cyprus, which we argue is essential in order to investigate the impact women’s health diseases have on HRQOL.

## Supplementary Information

Below is the link to the electronic supplementary material.

Supplementary file1 (XLSX 87 kb)

## Data Availability

The datasets generated and/or analysed during the current study will be published in open access journals and anonymous data will be available from the corresponding author on reasonable request.
